# Extraction and Back-Extraction Behaviors of La(III), Ce(III), Pr(III), and Nd(III) Single Rare Earth and Mixed Rare Earth by TODGA

**DOI:** 10.3390/s21248316

**Published:** 2021-12-12

**Authors:** Lina Qiu, Jiandi Li, Weiwei Zhang, Aijun Gong, Xiaotao Yuan, Yang Liu

**Affiliations:** 1 School of Chemistry and Biological Engineering, University of Science and Technology Beijing, Beijing 100083, China; qiulina@ustb.edu.cn (L.Q.); 18811345878@163.com (J.L.); yuanxt@ustb.edu.cn (X.Y.); s20200837@xs.ustb.edu.cn (Y.L.); 2 Beijing Key Laboratory for Science and Application of Functional Molecular and Crystalline Materials, University of Science and Technology Beijing, Beijing 100083, China; zhangweiwei@ustb.edu.cn; 3 Basic Experimental Center for Natural Science, University of Science and Technology Beijing, Beijing 100083, China

**Keywords:** light rare earths, extraction, back-extraction, TODGA, rate of recovery

## Abstract

*N*,*N*,*N*′,*N*′-Tetraoctyl diglycolamide (TODGA), as a new extraction agent, is effective for its excellent performance and low environmental hazard, and it is very welcome for the rare earth separation process. In this paper, by controlling the extraction time, diluent type, acid type and its concentration, rare earth concentration, etc., the optimum extraction and back-extraction effects of TODGA on La(III), Ce(III), Pr(III), and Nd(III) and mixed rare earths were obtained. The experiment showed that 0.10 mol·L^−1^ TODGA had the best extraction effect on single rare earth under the conditions of using petroleum ether as diluent, 5 mol·L^−1^ nitric acid, 20 min extraction time, and 0.01 mol·L^−1^ rare earth. In the mixed rare earth extraction, the percentage concentrations of La(III), Ce(III), Pr(III), and Nd(III) could be achieved from 21.7%, 19.9%, 30.8%, and 22.2% at the initial stage to 90.5%, 37%, 51%, and 62% after extraction, respectively, by controlling the number of back-extraction cycles and the concentrations of hydrochloric acid and nitric acid in the back-extraction system. The TODGA–rare earth carrier system showed the best back-extraction effect when the hydrochloric acid concentration was 1 mol·L^−1^ and the back-extraction time was 20 min. At the same time, the mixed rare earth liquid system with low initial concentration was selected for extraction and separation of mixed rare earth. The separation effect was better, and the recovery rate was higher than that of mixed rare earth liquid system with a high initial concentration.

## 1. Introduction

The rare earth elements can be grouped as light rare earth elements (La, Ce, Pr, Nd) and middle and heavy rare earth elements (Sm, Eu, Gd, Tb, Dy, Ho, Er, Tm, Yb, Lu, Y). Sc is a dispersed element, and Pm does not exist in nature [1]. Light rare earth elements (LREEs) are the most widely used elements in the application of rare earth; the production of light rare earth is about 75% of the total production [2]. Because of its special physical and chemical properties, it is widely used in the agriculture, battery, petrochemical, metallurgy, machinery, luminescent material, hydrogen storage material, environmental protection, medicine, and other fields [3,4,5,6,7,8,9]. Such rare earths are wildly used in sensors. Takuya’s group [10] conducted a comprehensive operando characterization of CO_2_ sensing with sensors based on hexagonal La_2_O_2_CO_3_. Christina [11,12] studied the application of Yi doped nanomaterials in high-temperature hydrogen sensors and found that Yi doped improved the sensitivity of the sensor. However, due to their similar chemical and physical properties, the separation of rare earths has been a problem that puzzled people for decades. Among all the separation methods studied, solvent extraction is considered to be the most desirable [13].

The amide pod ether extraction agent is a new environmental protection extractant developed in recent years. The cost of this kind of extractant is relatively low, and its degradation products are environmentally friendly and do not produce secondary pollution. It is a promising extractant [14,15]; one example, TODGA, has a good extraction ability for trivalent rare earth ions. In recent years, there have been many related studies on the extraction of some REEs, metal elements, and alkali metal elements using TODGA [16,17,18,19,20,21,22,23]. Sasaki [24] studied the extraction behavior of various metal ions from nitric acid medium using TODGA. Tachimori [25] explored the conditions of TODGA loading capacity. Ansari [26] studied the extraction thermodynamic behavior of TODGA and found that this process is exothermic. Ellis [27] found that TODGA was also able to distinguish adjacent LREEs.

In this paper, the extraction and back-extraction of La(III), Ce(III), Pr(III), and Nd(III) using TODGA under different parameters and the optimum conditions for the application of TODGA in mixed rare earth extraction were explored. The extraction rate was higher, and the effect of the light rare earth separation was better. The purpose of this study is to provide basic research data for the industrial use of TODGA; it is of great significance for the development of clean production processes of rare earth.

## 2. Experimental

### 2.1. Materials and Experimental Instruments

**Figure 1:**
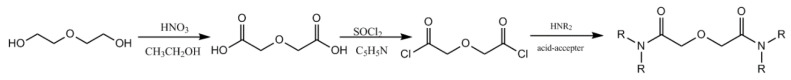
Synthetic process of TODGA.

TODGA was synthesized by our laboratory; the synthetic process is shown in Figure 1. The purity (>99%) was checked using NMR. Rare earth ions were determined by Agilent 7500ICP-MS.

### 2.2. Study on Extraction and Elution Characteristics of Single LREEs

The extraction and elution characteristics of four single rare earth ions were studied. The TODGA extractant and the organic phase after TODGA extraction were characterized by infrared spectrum.

#### 2.2.1. Extraction Characteristics of LREEs

(1) Effects of nitric acid concentrations and diluent types on extraction

Aqueous phases were 0.01 mol·L^−1^ of four kinds of LREEs in 0–5 mol·L^−1^ HNO_3_. Organic phases were 0.1 mol·L^−1^ TODGA in petroleum ether, *N*-octane, *N*-dodecane, or a mixture of *N*-octane and *N*-octanol with different ratios. Then, 10 mL of organic and aqueous phases were mixed in 100 mL screw cap glass vials and shaken for 20 min on an orbital vortex shaker at 2500 rpm and 20 °C.

(2) Hydrochloric acid extraction

On the basis of the previous conclusion, petroleum ether was selected as the diluent. Aqueous phases were 0.01 mol·L^−1^ of four kinds of LREEs in 1–6 mol·L^−1^ HCl. Organic phases were 0.1 mol·L^−1^ TODGA in petroleum ether. Then, 10 mL of organic and aqueous phases were mixed in 100 mL screw cap glass vials and shaken for 20 min on an orbital vortex shaker at 2500 rpm and 20 °C.

(3) Time dependent extraction

Aqueous phases were 0.005 mol·L^−1^ of four kinds of LREEs in 5 mol·L^−1^ HNO_3_. Organic phases were 0.1 mol·L^−1^ TODGA in petroleum ether. Then, 10 mL of organic and aqueous phases were shaken for 0, 5, 10, 20, 30, 40, and 50 min.

(4) Effects of different rare earth concentrations on extraction

The best extraction time and diluent were obtained on the basis of the previous results. The concentrations of HNO_3_ and TODGA, as well as the extraction temperature, remained unchanged. The initial concentrations of single rare earth were 0.005–0.03 mol·L^−1^.

#### 2.2.2. Back-Extraction Characteristics of LREEs

(1) Hydrochloric acid extraction

The TODGA system enriched with LREEs was selected as the organic phase. The back-extraction time was 20 min, and the concentrations of HCl were 0–9 mol·L^−1^. Equal amounts of aqueous and organic phase were shaken for 20 min at 2500 rpm and 20 °C.

(2) Time-dependent elution

HCl concentration was selected on the basis of the previous conclusion. The TODGA system enriched with LREEs was selected as organic phase. Equal amounts of aqueous and organic phase were shaken for 0, 5, 10, 20, 30, 40, and 50 min at 2500 rpm and 20 °C.

(3) Phosphoric acid and acetic acid elution

The TODGA system enriched with LREEs was selected as the organic phase. The concentrations of phosphoric acid in the aqueous phase were 0.00, 0.05, 0.10, 0.50, 1.00, 1.50, 2.00, 3.00, 4.00, and 5.00 mol·L^−1^. The concentrations of acetic acid in the aqueous phase were 0.00, 2.00, 5.00, 7.00, 9.00, and 10.00 mol·L^−1^. Phosphoric acid and acetic acid were eluted separately. Equal amounts of aqueous and organic phase were shaken for 20 min at 2500 rpm and 20 °C.

(4) Mixed hydrochloric acid and nitric acid back-extraction

The concentration of hydrochloric acid in the aqueous phase was 1.00 mol·L^−1^. The concentrations of nitric acid were 0.00, 0.05, 0.10, 0.50, 1.00, 1.50, 2.00, 3.00, 4.00, and 5.00 mol·L^−1^. Equal amounts of aqueous and organic phase were shaken for 20 min at 2500 rpm and 20 °C.

### 2.3. Extraction and Separation Characteristics of Mixed LREEs

The single rare earth in LREE mixture was extracted using TODGA. The conditions of extraction time, acid type, diluent, rare earth concentrations, etc. were studied to explore the optimum extraction and back-extraction by controlling the variables. The best conditions were applied to study the extraction and separation characteristics of mixed rare earth using TODGA.

#### 2.3.1. Preparation of Loading Rare Earth System

Equal volumes of the aqueous phase and the organic phase were used, in which the aqueous phase had four single rare earth elements in the low-concentration rare earth mixture of about 0.0025 mol·L^−1^ and in the high-concentration rare earth mixture of about 0.01 mol·L^−1^; the organic phase was TODGA solution with the concentration of petroleum ether diluent of 0.10 mol·L^−1^. At 20 °C and after 20 min of oscillation, the organic phase after treatment was a TODGA system containing La(III), Ce(III), Pr(III), and Nd(III) mixed rare earth. The aqueous phase before and after treatment was taken to analyze the concentration of rare earth ions. The TODGA system with low and high concentrations of La(III), Ce(III), Pr(III), and Nd(III) mixed rare earth was obtained.

#### 2.3.2. Effect of LREE Concentration on Separation Effect of Mixed Rare Earth

**Figure 2:**
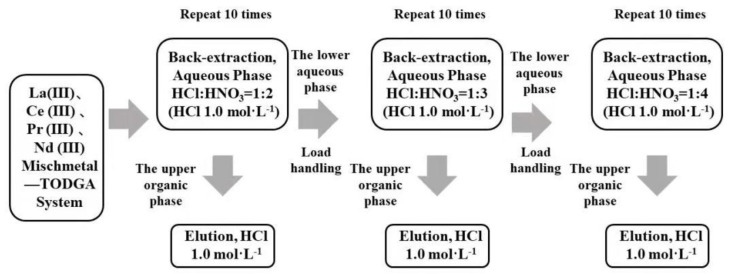
Flow chart of LREE separation in aqueous phase system by back-extraction using a mixed solution of hydrochloric acid and nitric acid.

TODGA containing the LREE mixture was back-extracted in three minor cycles. The separation flow chart of high-concentration and low-concentration rare earth in the aqueous phase system using a mixed solution of hydrochloric acid and nitric acid is shown in Figure 2. Equal amounts of aqueous and organic phase were shaken for 20 min at 2500 rpm and 20 °C. After every minor cycle, the LREE concentrations of the solution before and after loading, as well as the aqueous phase and organic phase after elution, were all determined, and the separation effect of elution and the single LREE recovery rate were compared.

#### 2.3.3. Back-Extraction of Low-Concentration Rare Earth in Aqueous Phase and Organic Phase

**Figure 3:**
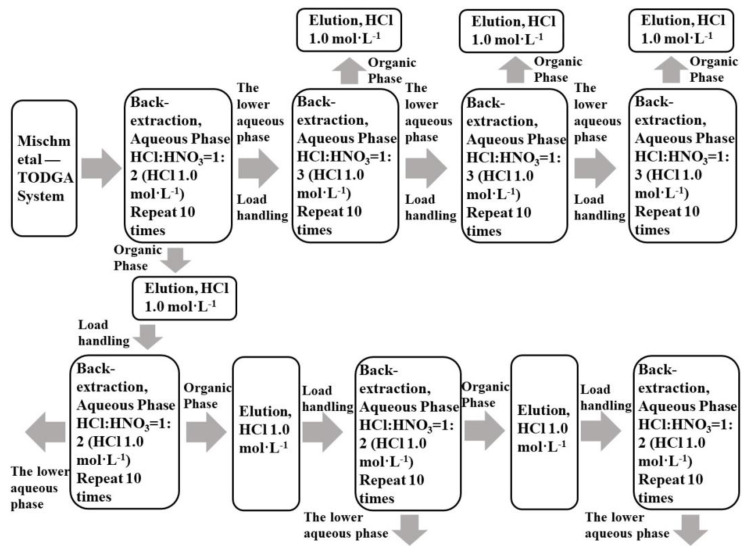
Flow chart of LREE separation in aqueous phase system and organic phase by back-extraction using a mixed solution of hydrochloric acid and nitric acid.

Mixtures of La(III), Ce(III), Pr(III), and Nd(III) with single rare earth concentrations of 0.0025 mol·L^−1^ were taken, and TODGA was loaded in accordance with Section 2.3.1. TODGA containing the LREE mixture was back-extracted in three minor cycles. The separation flow chart is shown in Figure 3. The mixed solution of hydrochloric acid and nitric acid was used to reverse extract low-concentration rare earth in the aqueous phase system.

## 3. Results and Discussion

### 3.1. Effects of Nitric Acid Concentrations and Diluent Types on Extraction Using TODGA

**Figure 4:**
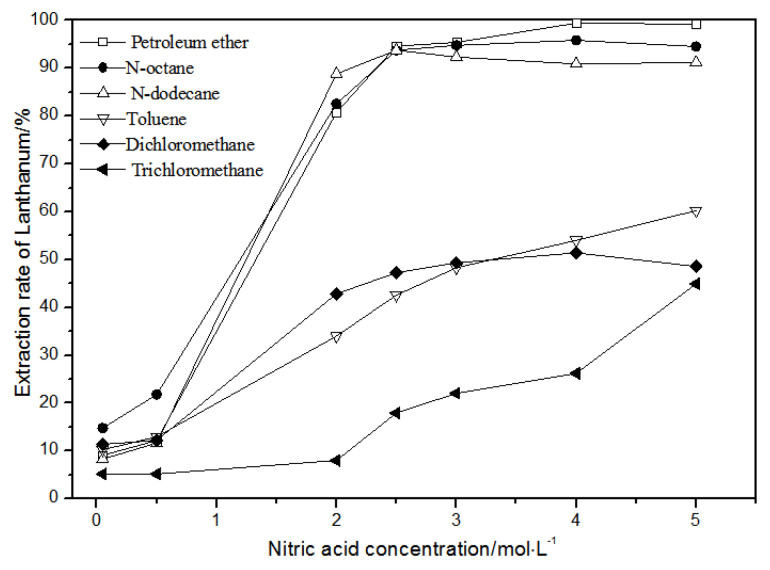
Effect of different nitric acid concentrations and different diluents on the extraction of La(III) by TODGA.

**Figure 5:**
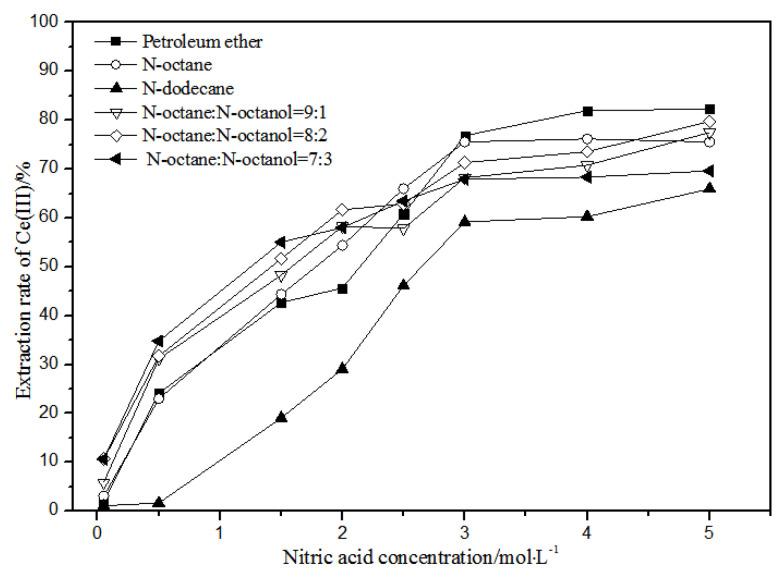
Effect of different nitric acid concentrations and different diluents on the extraction of Ce(III) by TODGA.

**Figure 6:**
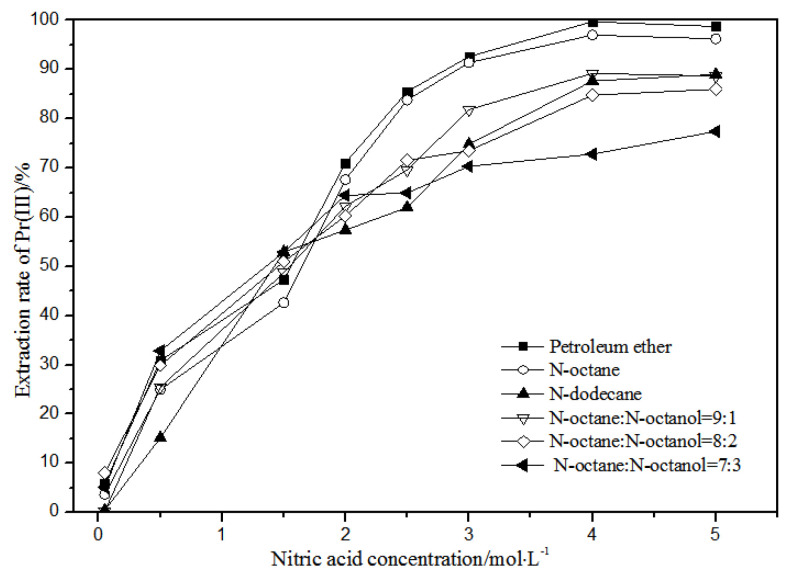
Effect of different nitric acid concentrations and different diluents on the extraction of Pr(III) by TODGA.

**Figure 7:**
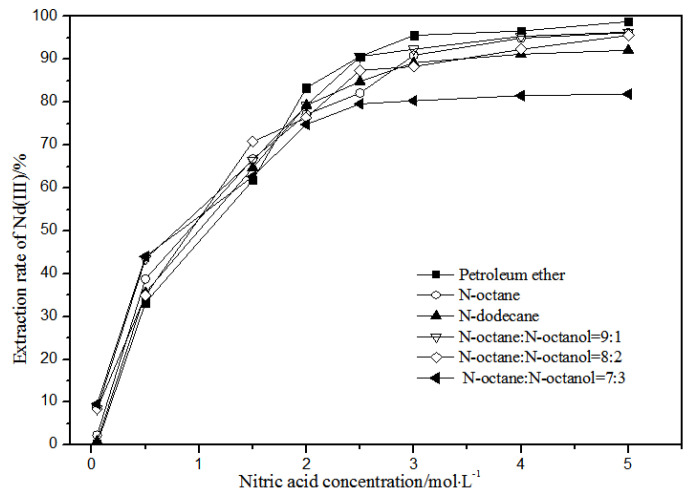
Effect of different nitric acid concentrations and different diluents on the extraction of Nd(III) by TODGA.

The effects of different nitric acid concentrations and diluents on the extraction of La(III), Ce(III), Pr(III), and Nd(III) using TODGA are shown in Figure 4, Figure 5, Figure 6 and Figure 7.

Comparing Figure 4, Figure 5, Figure 6 and Figure 7, the extraction rates of La(III), Ce(III), Pr(III), and Nd(III) increased slowly with nitric acid concentrations in the range of 0.05–1.5 mol·L^−1^. They increased rapidly from 1.5–3 mol·L^−1^ and reached the maximum extraction rate from 3–5 mol·L^−1^. Thus, when the extraction concentration of nitric acid was 5 mol·L^−1^, the extraction rates of La(III), Ce(III), Pr(III), and Nd(III) could reach the maximum in different diluent systems.

As shown in Figure 4, when toluene, dichloromethane, and chloroform were used as TODGA diluents and the nitric acid concentration was 5 mol·L^−1^, the extraction rate of La(III) by TODGA was only about 50%. Compared with the extraction rate of petroleum ether, *N*-octane, and *N*-dodecane under the same conditions, the extraction efficiencies of toluene, dichloromethane, and chloroform were not ideal. As the four LREEs have similar properties, according to previous studies [28], toluene, methylene chloride, and chloroform are not fit for the TODGA extraction system as diluents. Toluene, dichloromethane, and chloroform were separately replaced by organic phases with the volume ratio of *N*-octane to *N*-octanol 9:1, *N*-octane to *N*-octanol 8:2, and *N*-octane to *N*-octanol 7:3. According to the experimental results, it can be obtained that the extraction effect of La(III) is best with petroleum ether as the diluent of TODGA under the condition of 5 mol·L^−1^ nitric acid concentration.

According to Figure 5, under the condition of 5 mol·L^−1^ nitric acid, the extraction rate of Ce(III) with petroleum ether as the TODGA diluent was close to 82%, which is better than that with *N*-octane and *N*-dodecane as the diluents in the same conditions. When the volume ratios of *N*-octane to *N*-octanol were 9:1, 8:2, and 7:3, and the concentration of nitric acid was 5 mol·L^−1^, the extraction effect of 8:2 *N*-octane to *N*-octanol by volume ratio was the best among the three, but the effect was still not as good as that of petroleum ether as a diluent. It can be obtained that the extraction effect of Ce(III) was best with petroleum ether as the diluent of TODGA under the condition of 5 mol·L^−1^ nitric acid. From Figure 6 and Figure 7, when petroleum ether was used as the TODGA diluent, the extraction rate of Pr(III) was close to 100%, and that of Nd(III) was close to 99%.

Hence, La(III), Ce(III), Pr(III), and Nd(III) could be extracted with petroleum ether as the diluent for further experiments under the condition of 5 mol·L^−1^ nitric acid.

#### 3.1.1. Effects of Hydrochloric Acid Concentrations on Extraction Using TODGA

**Figure 8:**
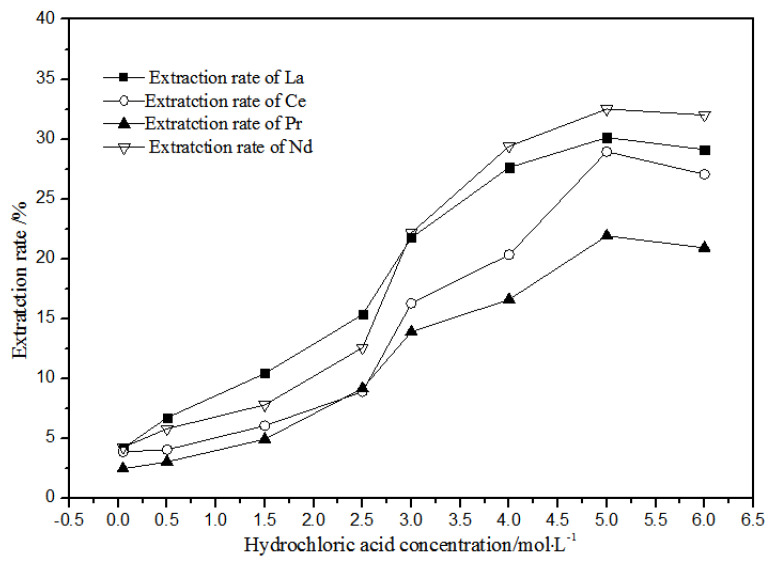
Effect of different hydrochloric acid concentrations on the extraction of La(III), Ce(III), Pr(III), and Nd(III) using TODGA.

As shown in Figure 8, when the concentration of hydrochloric acid was less than 5 mol·L^−1^, the extracted and distributed ratios of La(III), Ce(III), Pr(III), and Nd(III) in the TODGA-petroleum ether system increased gradually. When the concentration was 5 mol·L^−1^, the extraction and distribution ratios reached the maximum. When the concentration was greater than 5 mol·L^−1^, the extraction and distribution ratios reduced slightly. Under the condition of higher acid concentration, the extraction rate of hydrochloric acid extraction system could increase to 32.5%, whereas the extraction rate could increase to 99.4% in the nitric acid system. Therefore, the hydrochloric acid system is not suitable for TODGA to extract the aqueous phase system of La(III), Ce(III), Pr(III), and Nd(III) rare earth ions.

#### 3.1.2. Effects of Extraction Time on Extraction Characteristics Using TODGA

**Figure 9:**
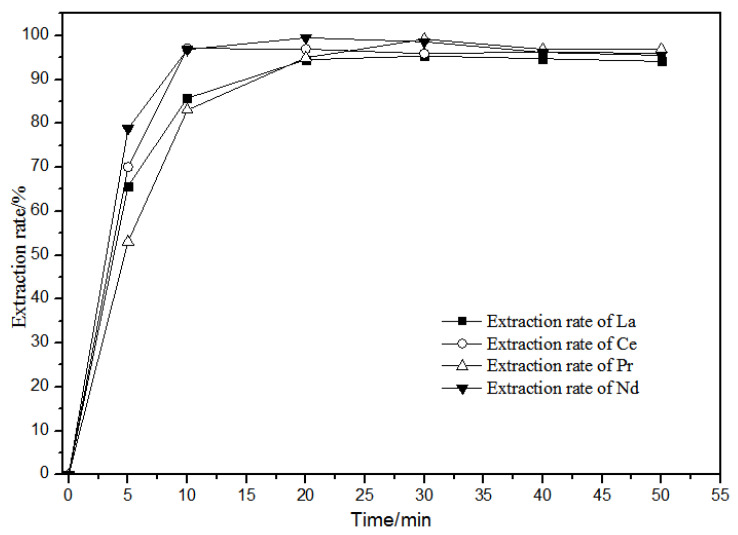
Effect of different extraction time on extraction characteristics of TODGA.

The extraction of La(III), Ce(III), Pr(III), Nd(III) in diluted petroleum ether at different extraction times of 0–50 min in the 0.10 mol·L^−1^ TODGA system is shown in Figure 9.

Figure 9 shows that, when the extraction time was over 20 min, the extraction rate in the aqueous phase reached the maximum and then tended to stabilize. La(III), Ce(III), Pr(III), and Nd(III) in the TODGA petroleum ether system had basically the same extraction behavior with time. Thus, the extraction reaction time is selected to be 20 min.

#### 3.1.3. Effects of Different Rare Earth Concentrations on Extraction

**Figure 10:**
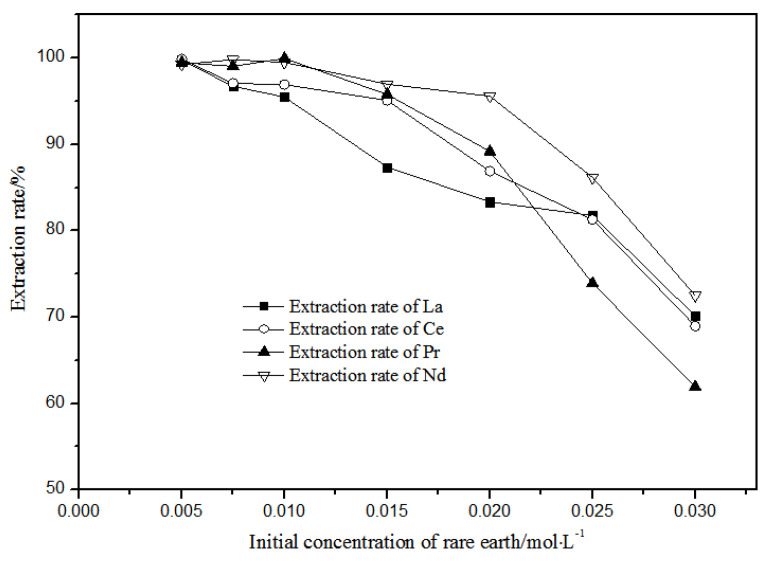
Effect of different concentrations of La(III), Ce(III), Pr(III), and Nd(III) on the extraction of La(III), Ce(III), Pr(III), and Nd(III) using TODGA.

With petroleum ether as diluent and 5 mol·L^−1^ nitric acid, the effects of single rare earth concentration on TODGA extraction efficiency in the range of 0.005–0.030 mol·L^−1^ were investigated, and the results are shown in Figure 10.

When the single rare earth concentration of La(III), Ce(III), Pr(III), and Nd(III) was less than or equal to 0.01 mol·L^−1^, the extraction and distribution ratios of La(III), Ce(III), Pr(III), and Nd(III) could all achieve more than 95% in the TODGA-petroleum ether system. When the single rare earth concentration of La(III), Ce(III), Pr(III), and Nd(III) was equal to 0.005 mol·L^−1^, the extraction and distribution ratios of the La(III), Ce(III), Pr(III), and Nd(III) in the TODGA-petroleum ether system were 96.75%, 97.09%, 99.06%, and 99.83%. When the single rare earth concentration of La(III), Ce(III), Pr(III), Nd(III) was greater than 0.01 mol·L^−1^, the extraction and distribution ratios of La(III), Ce(III), Pr(III), and Nd(III) were gradually reduced in the TODGA-petroleum ether system. When the concentration of each single rare earth was 0.03 mol·L^−1^, the extraction and distribution ratios of La(III), Ce(III), Pr(III), and Nd(III) in the TODGA–petroleum ether system were 70.13%, 68.96%, 61.93%, and 72.51%. Selecting a system with high extraction rate will be more conducive to the separation and purification of rare earths. At the same time, considering the extraction efficiency, La(III), Ce(III), Pr(III), and Nd(III) with 0.01 mol·L^−1^ single rare earth concentration were selected in the aqueous system for extraction.

#### 3.1.4. IR Spectrum of Organic Phase after Extraction Using TODGA

**Figure 11:**
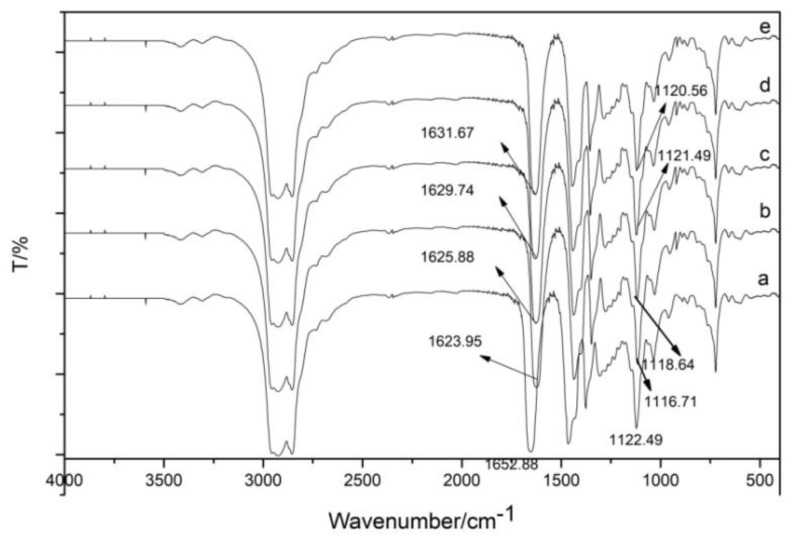
The infrared spectra of TODGA extractant and the organic phase after extraction using TODGA of trivalent rare earth ions La(III), Ce(III), Pr(III), and Nd(III): (a) TODGA; (b) La-TODGA; (c) Ce-TODGA; (d) Pr-TODGA; (e) Nd-TODGA.

TODGA and the extracted organic phase are liquid. Therefore, the KBr film coating method was used, and the Shimadzu Fourier-transform infrared spectrometer FTIR-8400S infrared spectrometer was used to collect infrared spectra of substances in the range of 4000–400 cm^−1^. The number of scans was set to four, and the resolution was set to 2 cm^−1^. Figure 11 shows the infrared spectrum of TODGA extraction agent and the organic phase after TODGA extraction of trivalent rare earth ions La(III), Ce(III), Pr(III), and Nd(III). The peak appearing at the position of 1652 cm^−1^ corresponds to the carbonyl C=O stretching vibration. The corresponding bond C-O-C at 1122 cm^−1^ is antisymmetric stretching vibration. TODGA was used to extract La(III), Ce(III), Pr(III), and Nd(III). After the extraction, the absorption peaks at 1652 cm^−1^ and 1122 cm^−1^ were red-shifted to a lower wave number, indicating that the carbonyl C=O and ether bond C-O-C were coordinated with La(III), Ce(III), Pr(III), and Nd(III).

### 3.2. Back-Extraction Characteristics of Rare Earth Using TODGA

#### 3.2.1. Effects of Hydrochloric Acid Concentrations on Back-Extraction Using TODGA

**Figure 12:**
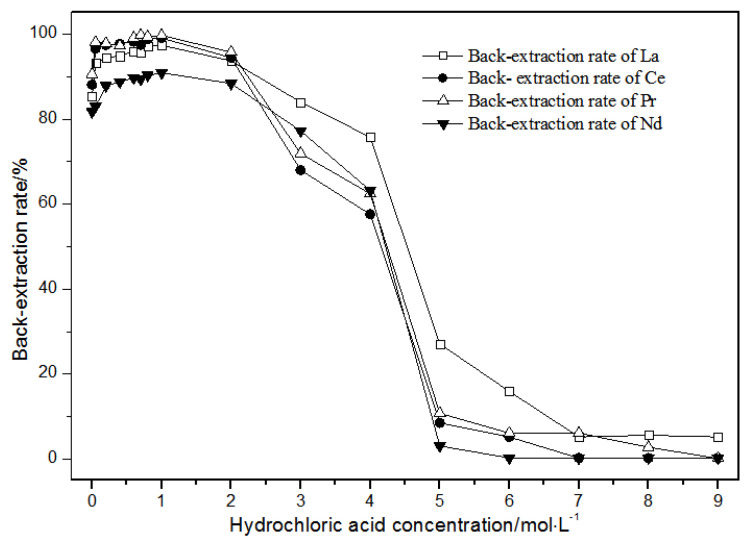
Effect of concentrations of hydrochloric acid on La(III), Ce(III), Pr(III), and Nd(III) back-extraction.

The TODGA system enriched with La(III), Ce(III), Pr(III), and Nd(III) was selected as the organic phase. The extraction time was 20 min. The effect of the hydrochloric acid concentrations on back-extraction is shown in Figure 12.

Under the condition that the hydrochloric acid concentration was 1 mol·L^−1^ or less, the single rare earth La(III), Ce(III), Pr(III), and Nd(III) back-extraction rate gradually increased with the hydrochloric acid concentration. When the hydrochloric acid concentration was 1 mol·L^−1^, the single rare earth elution rates in the TODGA system reached the maximum. When the hydrochloric acid concentration was between 1 and 2 mol·L^−1^, the single rare earth back-extraction rates of La(III), Ce(III), Pr(III), and Nd(III) were slightly reduced. When the concentration of hydrochloric acid was between 2 and 5 mol·L^−1^, the single rare earth back-extraction rates of La(III), Ce(III), Pr(III), and Nd(III) were greatly reduced. When the hydrochloric acid concentration was more than 5 mol·L^−1^, the single rare earth back-extraction rates remained basically unchanged, and the elution effect was not ideal. Thus, the optimal hydrochloric acid concentration was 1 mol·L^−1^, where the extraction rate reached the maximum, and the elution effect was the best.

#### 3.2.2. Effects of Time on the Back-Extraction

**Figure 13:**
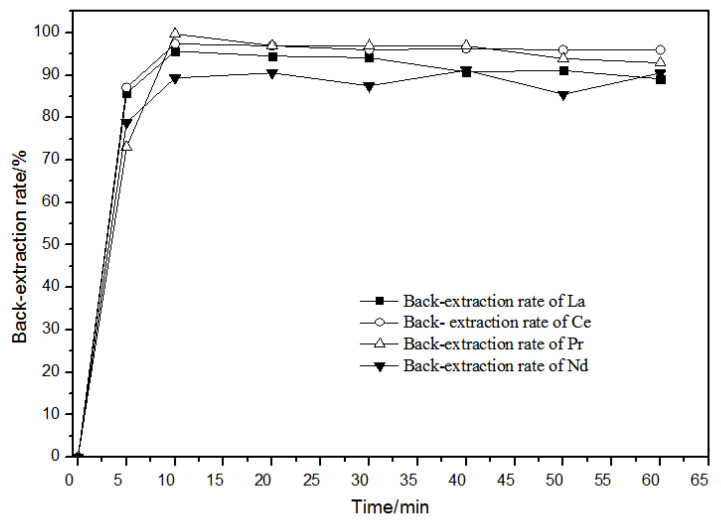
Effect of different times on La(III), Ce(III), Pr(III), and Nd(III) back-extraction.

When the hydrochloric acid concentration in the aqueous phase was 1.0 mol·L^−1^, the effect of time on back-extraction was as shown in Figure 13. When the back-extraction time was 10 min, the back-extraction rates of rare earth ions remained unchanged or even slightly declined. Over time, the back-extraction behaviors of La(III), Ce(III), Pr(III), and Nd(III) ions were basically the same. For this reason, the back-extraction time was selected as 20 min.

#### 3.2.3. Effects of the Concentrations of Phosphate Acid and Acetic Acid on Back-Extraction

**Figure 14:**
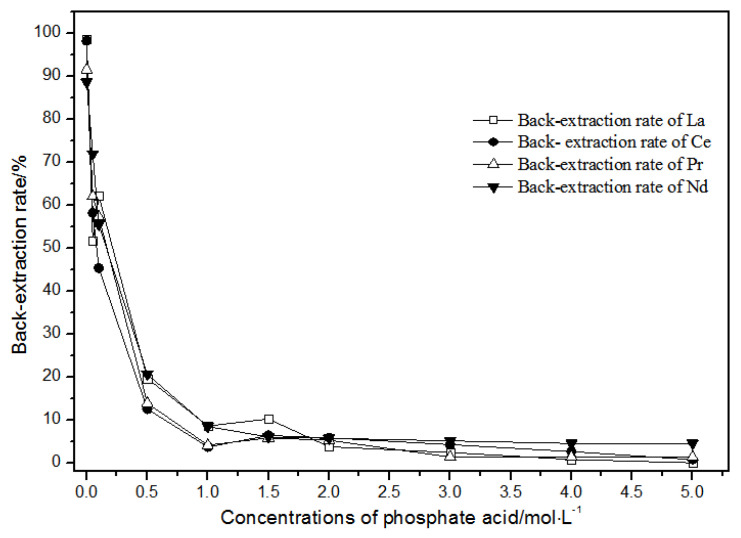
Effect of different concentrations of phosphate acid on La(III), Ce(III), Pr(III), and Nd(III) back-extraction.

**Figure 15:**
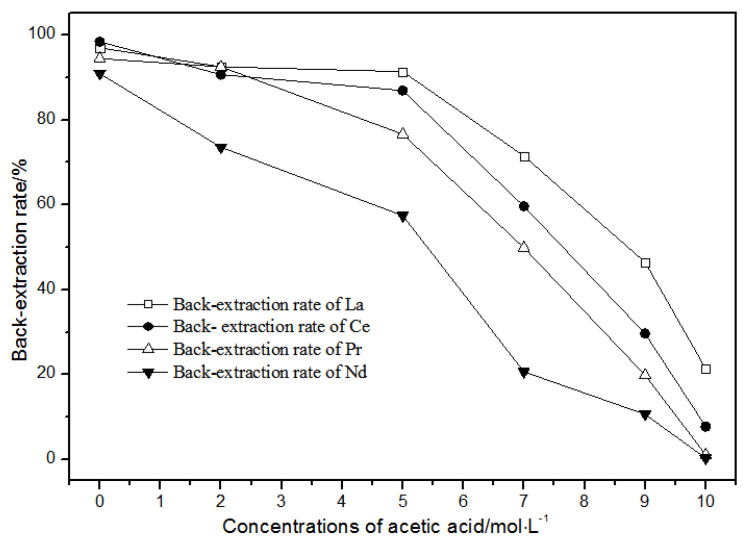
Effect of different concentrations of acetic acid on La(III), Ce(III), Pr(III), and Nd(III) back-extraction.

The back-extraction (Figure 14) results show that the single rare earth back-extraction rate at 1.00 mol·L^−1^ phosphate acid was the highest compared to other concentrations. However, the extraction effect was still not ideal. Using different acetic acid concentrations (Figure 15), the single rare earth back-extraction rates decreased with the increase in acetic acid concentration. Compared with 1.00 mol·L^−1^ hydrochloric acid, the back-extraction rates of both kinds of acid were not ideal.

#### 3.2.4. Effects of Concentrations of Mixed Hydrochloric Acid and Nitric Acid on Back-Extraction

**Figure 16:**
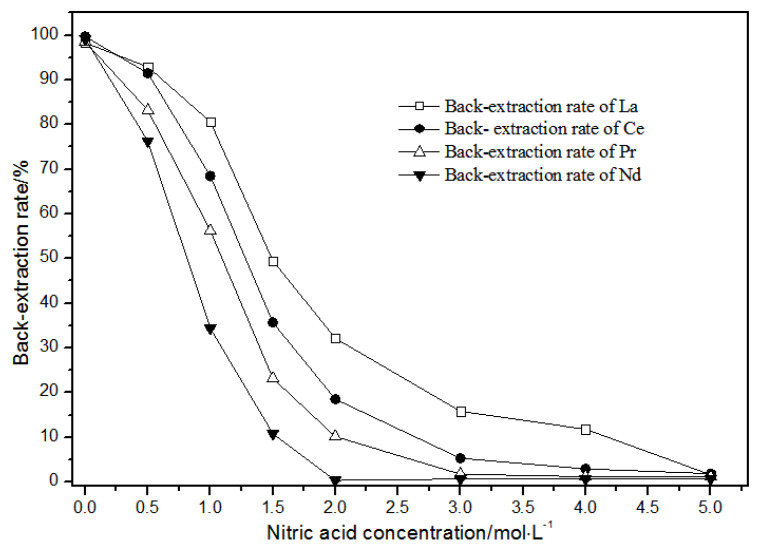
Effect of concentrations of mixed hydrochloric acid and nitric acid on La(III), Ce(III), Pr(III), and Nd(III) back-extraction.

The TODGA system enriched with La(III), Ce(III), Pr(III), and Nd(III) was selected as the organic phase. The extraction time was 20 min, and the aqueous phase was a mixture of hydrochloric acid and nitric acid. The effect of the concentrations of mixed hydrochloric acid and nitric acid on back-extraction is shown in Figure 16.

As the nitric acid concentration in the mixture of hydrochloric acid and nitric acid gradually increased (Figure 16), the single rare earth back-extraction rate of LREEs gradually decreased. The back-extraction rates of LREEs showed gradient changes in the mixture of 1.00 mol·L^−1^ hydrochloric acid and 0.05 mol·L^−1^ nitric acid.

The distribution ratios were different in these conditions. In the mixture of 1.00 mol·L^−1^ hydrochloric acid and 2.00 mol·L^−1^ nitric acid, single rare earth La(III), Ce(III), and Pr(III) had higher back-extraction rates, while the back-extraction rate of Nd(III) was close to 0. In the mixture of 1.00 mol·L^−1^ hydrochloric acid and 3.00 mol·L^−1^ nitric acid, single rare earth La(III) and Ce(III) had higher back-extraction rates, while the back-extraction rates of Pr(III) and Nd(III) were close to 0. In the mixture of 1.0 mol·L^−1^ hydrochloric acid and 4.00 mol·L^−1^ nitric acid, the back-extraction rate of La(III) was more than 10%, while the back-extraction rates of Pr(III), Nd(III), and Ce(III) were close to 0.

Thus, the separation of mixed rare earth could be achieved using a mixture of 1.0 mol·L^−1^ hydrochloric acid and 2.00–4.00 mol·L^−1^ nitric acid concentrations.

### 3.3. The Extraction and Separation of Mixed LREEs Using TODGA

#### 3.3.1. Effect of High-Concentration Rare Earth Mixture on Separation

(1) The first small cycle operation of the high-concentration rare earth mixed liquid system

**Figure 17:**
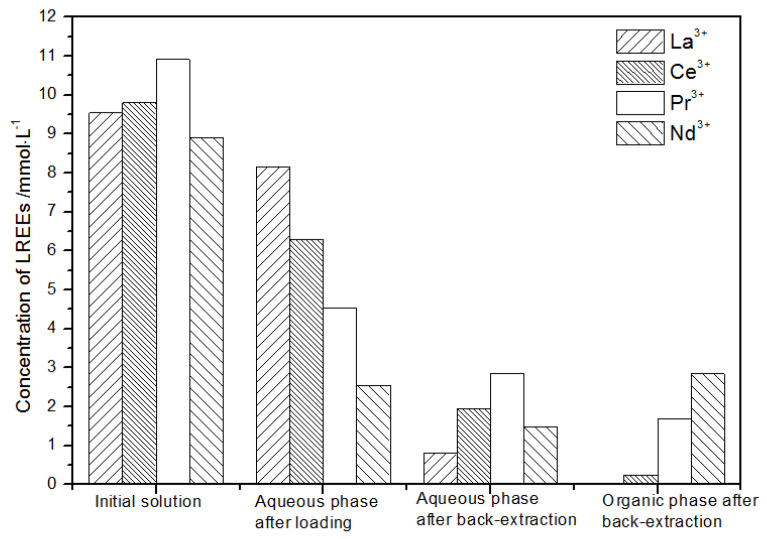
Change in concentration after the first small cycle of the high-concentration rare earth mixture system.

After loading treatment, the concentration of mixed rare earth in the aqueous phase was still high (Figure 17). This is because the initial concentration of the mixed rare earth was too high, indicating that the rare earth loading of TODGA reached the maximum. After back-extraction, the concentration of Pr(III) in the aqueous phase was the highest, while that of La(III), Ce(III), and Nd(III) was relatively low, but the separation was not obvious. The concentrations of Pr(III) and Nd(III) in the organic phase after back-extraction were significantly higher than those of La(III) and Ce(III), and the separation effect was obvious. However, the recovery rates of La(III), Ce(III), Pr(III), and Nd(III) in this cycle operation were 59.7%, 62.8%, 70.9%, and 67.8%, respectively. The recoveries were low, and the loss of rare earth was too large.

(2) The second small cycle operation of the high-concentration rare earth mixed liquid system

**Figure 18:**
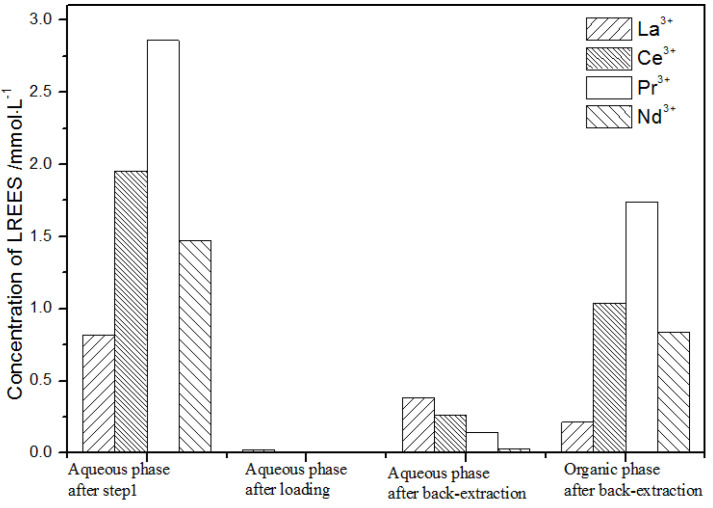
Change in concentration after the second small cycle of the high-concentration rare earth mixture system.

After the second small circulation operation of the high-concentration rare earth mixed liquid system, the mixed rare earth concentrations of the initial solution, the aqueous phase after loading, the water phase, and organic phase after elution were as shown in Figure 18.

The concentration of mixed rare earth in the aqueous phase after back-extraction in step (1) was extremely low, indicating that almost all rare earth ions were contained in the TODGA organic phase. After elution, the concentration of Pr(III) in the water phase was low, while that of La(III), Ce(III), and Nd(III) was relatively high. After the back-extraction, the concentration of Pr(III) in the organic phase eluent was detected. The concentration of Pr(III) in the concentrated solution was 45.4% of the total concentration of rare earth ions, which was significantly higher than that of La(III), Ce(III), and Nd(III), but the separation effect was not obvious. The recovery rates of La(III), Ce(III), Pr(III), and Nd(III) were 75.2%, 67.1%, 66.0%, and 59.1%, respectively. The loss of rare earth was excessive under these conditions.

(3) The third small cycle operation of the high-concentration rare earth mixed liquid system

**Figure 19:**
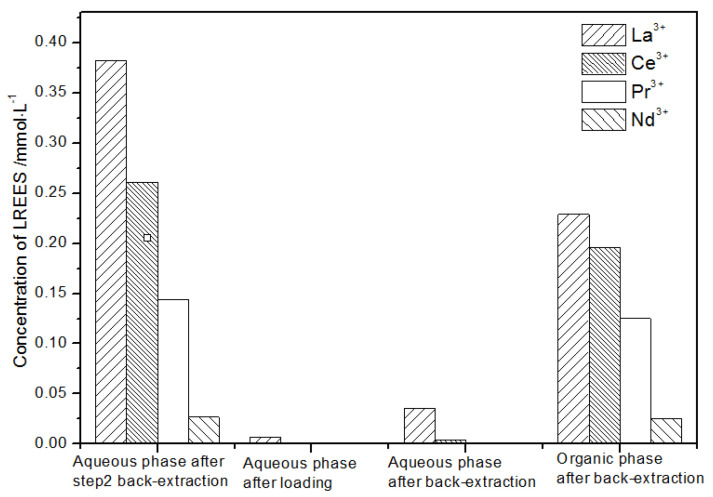
Concentration diagram after the third small cycle operation of the high-concentration rare earth mixed liquid system.

It can be seen from Figure 19 that, after loading treatment, the concentration of mixed rare earth in the aqueous phase after back-extraction in step (2) was extremely low, indicating that almost all the rare earth was contained in the TODGA organic phase. La(III) in the aqueous phase after elution was 87.9% of the total rare earth ion concentration, much higher than that of Ce(III), Pr(III), and Nd(III). After this operation, almost all other LREEs were separated. After elution operation, the concentration of Nd(III) in the organic phase after elution was relatively low, and the concentrations of La(III), Ce(III), and Pr(III) decreased in order, but the separation effect was not obvious. The recovery rates of La(III), Ce(III), Pr(III), and Nd(III) were 70.7%, 77.1%, 87.8%, and 96.7%, respectively. The loss of rare earth during operation was relatively small.

#### 3.3.2. Effect of Low-Concentration Rare Earth Mixture in Aqueous Phase on Separation

(1) The first small cycle operation of the low-concentration rare earth mixed liquid system

**Figure 20:**
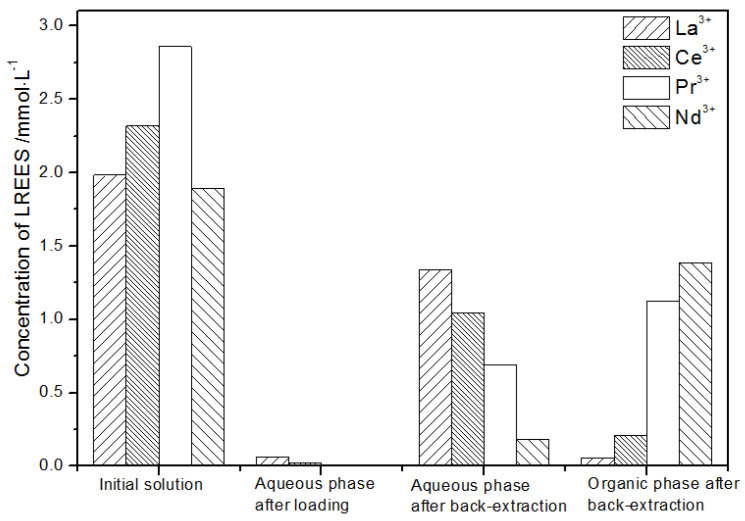
Concentration diagram after the first small cycle operation of the low-concentration rare earth mixed liquid system.

The initial concentration of the mixed rare earths in the first small cycle operation, the concentration of the mixed rare earths in the aqueous phase after loading, the concentration of the mixed rare earths in the aqueous phase after back-extraction, and the concentration of the mixed rare earths in the organic phase after back-extraction are shown in Figure 20.

It can be seen from Figure 20 that, after the initial mixed rare earths were loaded, almost all of the rare earths were loaded into the TODGA organic phase. The concentrations of La(III), Ce(III), and Pr(III) in the mixed rare earth in the aqueous phase after the elution were relatively high, and the concentration of Nd(III) was relatively low, but the separation was not obvious. After the elution, the concentrations of Pr(III) and Nd(III) in the organic phase were significantly higher than the concentrations of La(III) and Ce(III), accounting for 40.5% and 49.8% of the total rare earth ion concentration. The separation effect was obvious. The recovery rates of La(III), Ce(III), Pr(III), and Nd(III) single rare earths under this cycle operation were 72.9%, 54.9%, 63.9%, and 83.5%, respectively. The loss of rare earth was excessive.

(2) The second small cycle operation of the low-concentration rare earth mixed liquid system

**Figure 21:**
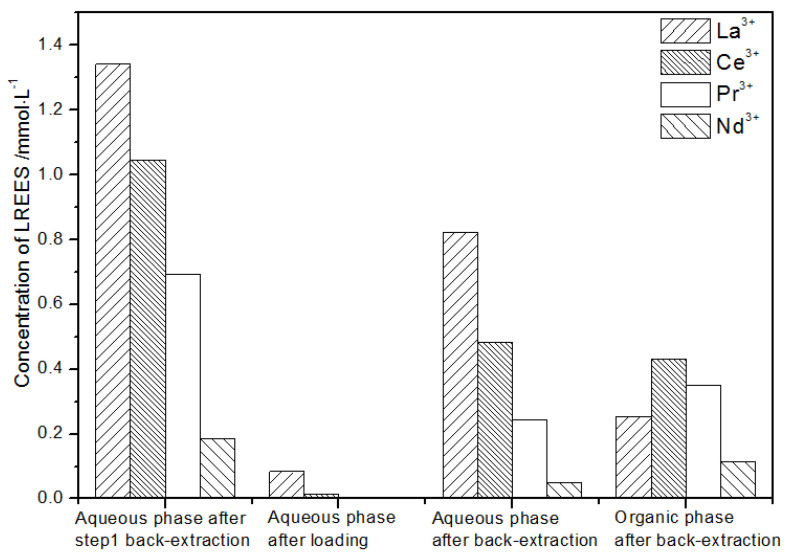
Concentration diagram of the second small cycle operation of the low-concentration rare earth mixed liquid system.

After the loading treatment, the concentration of the mixed rare earth in the aqueous phase after elution in step (1) was extremely low (Figure 21), indicating that almost all of the rare earth was contained in the TODGA organic phase. After back-extraction, the concentration of mixed rare earth Pr(III) in the aqueous phase was low, while that of La(III), Ce(III), and Nd(III) was relatively higher. After this cycle, Pr(III) was almost separated. The concentrations of La(III), Ce(III), and Pr(III) in the organic phase after back-extraction were slightly higher than that of Nd(III), but the separation effect was not obvious. The recovery rates of La(III), Ce(III), Pr(III), and Nd(III) single rare earths were 85.7%, 88.1%, 86.4%, and 89.8%, respectively. Therefore, the loss of rare earth was small during the experiment.

(3) The third small cycle operation of the low concentration rare earth mixed liquid system

**Figure 22:**
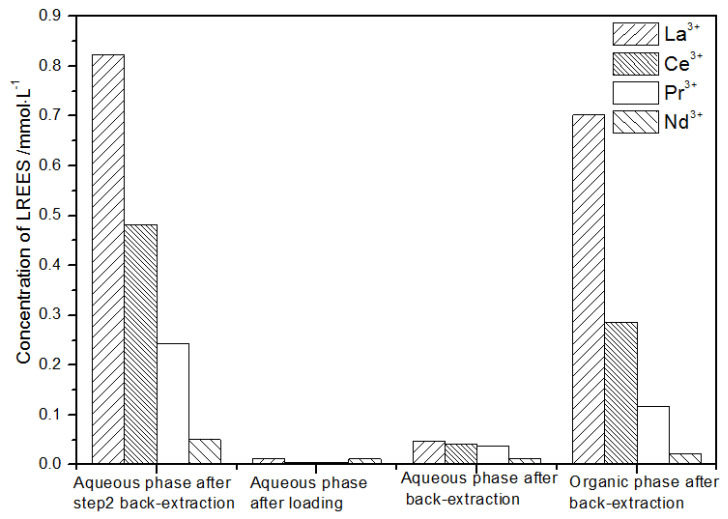
Concentration diagram of the third small cycle operation of the low-concentration rare earth mixed liquid system.

After loading (Figure 22), the concentration of the mixed rare earth in the aqueous phase after the elution operation in step (2) was extremely low, indicating that almost all of the rare earth was contained in the TODGA organic phase. After back-extraction, the concentration of Nd(III) in the aqueous phase was relatively low, while the concentrations of La(III), Ce(III), and Pr(III) decreased in order, but the separation effect was not obvious. The concentration of La(III) in the organic phase after elution was higher than that of Ce(III), Pr(III), and Nd(III). The concentration of La(III) accounted for 62.2% of the total rare earth concentration, and the separation effect was obvious. Under this cycle operation, the recovery rates of La(III), Ce(III), Pr(III), and Nd(III) were 92.4%, 68.8%, 64.1%, 89.4%, respectively. The recovery rates of Ce(III) and Pr(III) were both only about 65%; thus, the loss of rare earth was relatively large during this experiment. On the other hand, the recovery rates of La(III) and Nd(III) were about 90%, which shows that the loss during this cycle was relatively small.

#### 3.3.3. Reverse Extraction of Low-Concentration Rare Earth in Aqueous Phase and Organic Phase Using Hydrochloric Acid and Nitric Acid Mixture

(1) The first operation of the low-concentration rare earth mixed liquid system

**Figure 23:**
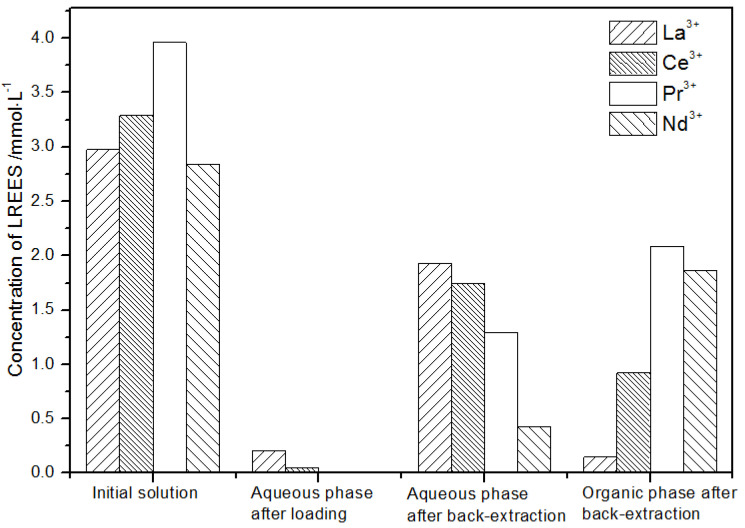
Concentration diagram of the first operation of the low-concentration rare earth mixed liquid system.

It can be seen from Figure 23 that, after the initial mixed rare earth was subjected to the loading treatment, almost all the rare earths were in the TODGA organic phase. The concentration of Nd(III) in the water phase after the back-extraction operation was relatively low, while the concentrations of La(III), Ce(III), and Pr(III) decreased in order, but the separation effect was not obvious. The concentrations of Pr(III) and Nd(III) in the organic phase after elution were obviously higher than the concentrations of La(III) and Ce(III), but the separation effect was not obvious. Due to the different initial rare earth concentrations, the rare earth amount in the organic phase after different low-concentration elution was different. However, the trends of the rare earth content in the aqueous phase after extraction were generally the same. The recovery rates of La(III), Ce(III), Pr(III), and Nd(III) were 75.0%, 82.2%, 85.5%, and 80.6%, respectively. The recovery rates of the four REEs were all around 80%. It can be seen that the loss of rare earth during the experiments was small.

(2) The first small cycle of the aqueous phase after the elution of low-concentration rare earth mixed liquid system

**Figure 24:**
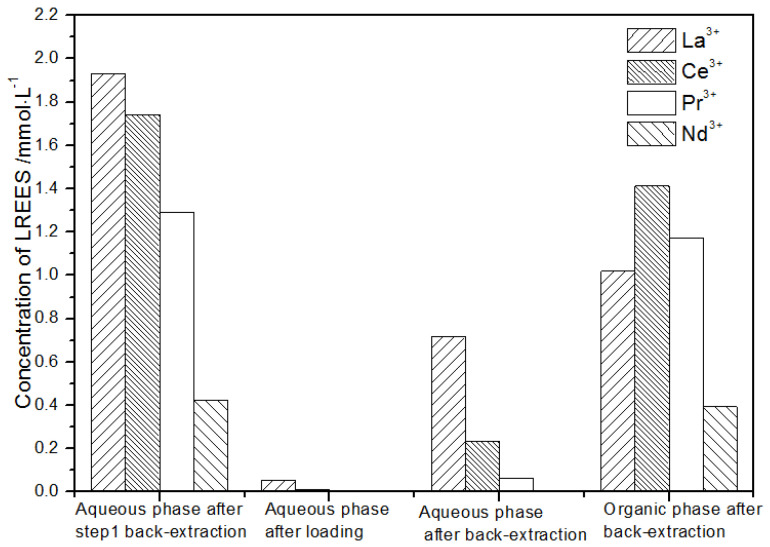
Concentration diagram of the first small cycle operation of the aqueous phase after elution of the low-concentration rare earth mixed liquid system.

It can be seen from Figure 24 that the aqueous phase after the elution of step (1) was subjected to a load treatment, and then the concentration of the mixed rare earth in the aqueous phase was extremely low, indicating that almost all the rare earth was contained in the TODGA organic phase. After back-extraction, the concentration Nd(III) in the aqueous phase was relatively low, while the concentrations of La(III), Ce(III), and Pr(III) decreased in order. The concentration of La(III) accounted for 70.4% of the total rare earth concentration. The concentrations of Ce(III) and Pr(III) accounted for 22.7% and 6.3% of the total rare earth concentration, respectively, and the separation effect was obvious. After back-extraction, the concentration of Nd(III) in the organic phase after elution was relatively low, and the concentrations of La(III), Ce(III), and Pr(III) decreased sequentially, but the separation effect was not obvious. The recovery rates of La(III), Ce(III), Pr(III), and Nd(III) single rare earths were 92.6%, 95.1%, 96.0%, and 94.2%, respectively. It can be seen that the loss of rare earth during the experiment was small.

(3) The second small cycle of the aqueous phase after elution of the low-concentration rare earth mixed liquid system

**Figure 25:**
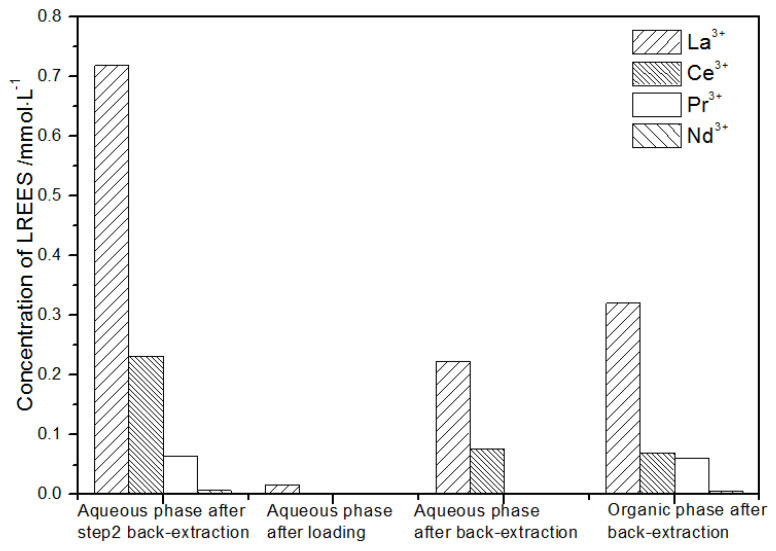
Concentration diagram of the second small cycle operation of the aqueous phase after elution of the low-concentration rare earth mixed liquid system.

The aqueous phase (see Figure 25) after elution of step (2) was subjected to a load treatment, and then the concentration of the mixed rare earth in the aqueous phase was extremely low, indicating that almost all the rare earth was contained in the TODGA organic phase. The concentration of La(III) in the aqueous phase was relatively high after back-extraction, while the concentrations of Ce(III), Pr(III), and Nd(III) were extremely low. The concentration of La(III) accounted for 74.1% of the total rare earth ion concentration. The concentrations of Ce(III), Pr(III), and Nd(III) accounted for 25.4%, 0.4%, and 0.1% of the total rare earth ion concentration, respectively. The separation effect of La(III) was obvious. The concentrations of Ce(III), Pr(III), and Nd(III) in the organic phase after elution were relatively low, while the concentration of La(III) was relatively high. The concentration of La(III) accounted for 70.1% of the total rare earth concentration, while the concentrations of Ce(III), Pr(III), and Nd(III) accounted for 15.2%, 13.5%, and 1.2% of the total rare earth concentration, respectively. The separation effect was obvious. The recovery rates of La(III), Ce(III), Pr(III), and Nd(III) single rare earths under this cycle operation were 77.4%, 63.3%, 98.2%, and 85.1%, respectively. The recovery rates of La(III) and Ce(III) were both about 70%, which shows that the loss was relatively large during the experiment. The recovery rates of Pr(III) and Nd(III) were both above 85%, which shows that the loss during the experiment was relatively small.

(4) The third small cycle of the aqueous phase after elution of the low concentration rare earth mixed liquid system

**Figure 26:**
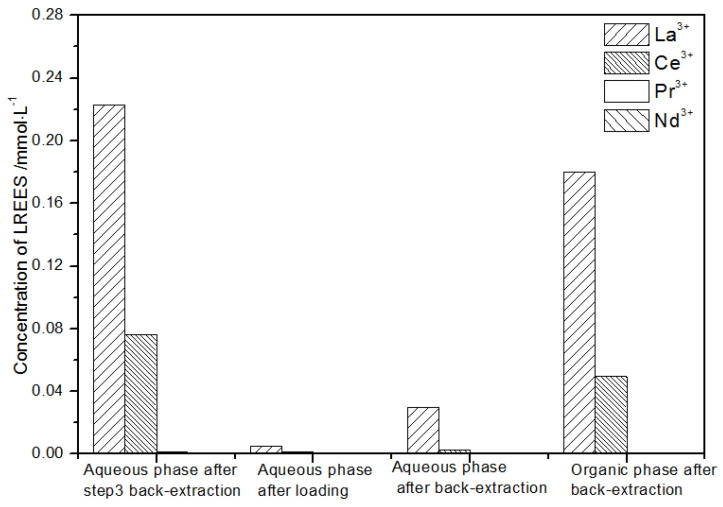
Concentration diagram of the third small cycle operation of the aqueous phase after elution of the low-concentration rare earth mixed liquid system.

It can be seen from Figure 26 that the concentration of mixed rare earth in the aqueous phase after elution in step (3) was very low after the aqueous phase was loaded, indicating that almost all the rare earth was contained in the TODGA organic phase. The concentration of La(III) in the aqueous phase was relatively high after elution, while the concentrations of Ce(III), Pr(III), and Nd(III) were extremely low. The concentration of La(III) accounted for 90.5% of the total rare earth concentration, while the concentrations of Ce(III), Pr(III), and Nd(III) accounted for 8.6%, 0.6%, and 0.3% of the total rare earth concentration, respectively. The separation effect of La(III) was obvious. After back-extraction, the concentration of La(III) in the organic phase after elution was relatively high, while the concentrations of Ce(III), Pr(III), and Nd(III) decreased in order. The concentration of La(III) accounting for the total rare earth concentration was 78.1%, while the concentrations of Ce(III), Pr(III), and Nd(III) accounted for 21.5%, 0.4%, and 0.02% of the total rare earth concentration, respectively. The separation effect was not as obvious as that of the water phase after elution. The recovery rates of La(III), Ce(III), Pr(III), and Nd(III) were 96.3%, 70.1%, 90.1%, and 92.0%, respectively. Thus, this step had relatively smaller loss; the recovery rate of Ce(III) was about 70%, whereas the recovery rates of La(III), Pr(III), and Nd(III) were all over 90%. The loss of rare earth during the experiment was relatively large.

(5) The first small cycle of the organic phase after elution of the low-concentration rare earth mixed liquid system

**Figure 27:**
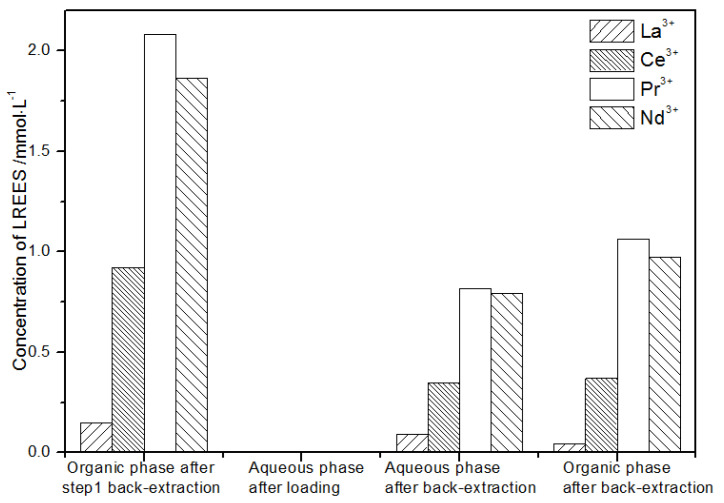
Concentration diagram of the first small cycle of the organic phase after low-concentration elution.

In the first small cycle of the organic phase after elution of the low-concentration rare earth mixed liquid system, the initial mixed rare earth concentration, the mixed rare earth concentration in the aqueous phase after loading, and the mixed rare earth concentration in the aqueous phase and organic phase after elution are shown in Figure 27.

It can be seen from Figure 27 that, after elution, concentration, and loading, the concentration of the mixed rare earth in the aqueous phase after the back-extraction in step (1) was extremely low, indicating that almost all of the rare earth was loaded in the organic phase.

After back-extraction, the concentrations of La(III), Ce(III), Nd(III), and Pr(III) in the aqueous phase increased in order. The concentrations of La(III) and Ce(III) accounted for 4.4% and 17.0%, while the concentrations of Pr(III) and Nd(III) accounted for 39.9% and 38.7% of the total rare earth ion concentration, respectively, but the separation effect was not obvious. The concentrations of La(III), Ce(III), Nd(III), and Pr(III) in the organic phase after elution increased in order. The concentrations of La(III) and Ce(III) accounting for the total concentration of rare earth ion were 1.9% and 15.1%, while the concentrations of Pr(III) and Nd(III) accounted for 43.3% and 39.7% of the total rare earth ion concentration, respectively, but the separation effect was not obvious. The recovery rates of La(III), Ce(III), Pr(III), and Nd(III) single rare earth under this cycle operation were 93.8%, 78.2%, 90.4%, and 94.9%, respectively, which shows that the loss during the experiment was relatively small.

(6) The second small cycle of the organic phase after elution of the low-concentration rare earth mixed liquid system

**Figure 28:**
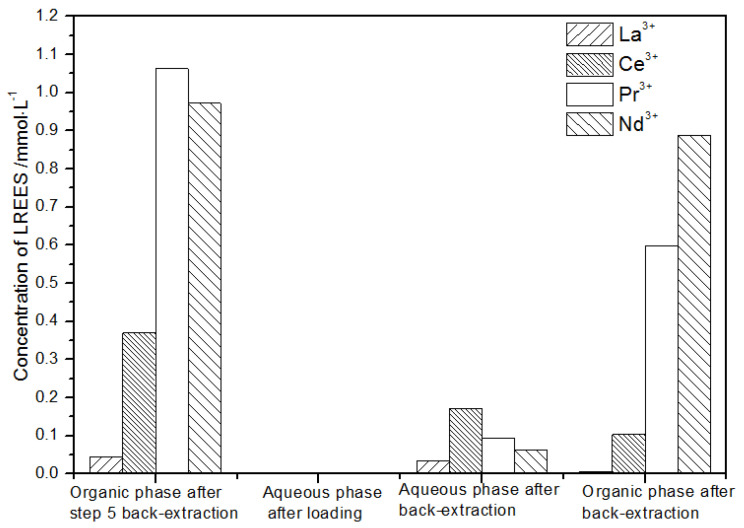
Concentration diagram of the second small cycle operation of the organic phase after elution of the low-concentration rare earth mixed liquid.

It can be seen from Figure 28 that, after elution, concentration, and loading, the concentration of the mixed rare earth in aqueous phase in step (5) was extremely low, indicating that almost all of the rare earth was contained in the TODGA organic phase. After back-extraction, the concentrations of Ce(III), Nd(III), and Pr(III) in the aqueous phase decreased in order, while the concentration of La(III) was relatively low. The concentration of La(III) accounted for 9.4% of the total rare earth concentration, while the concentrations of Ce(III), Pr(III), and Nd(III) accounted for 47.6%, 26.0%, and 17.1% of the total rare earth concentration, respectively, but the separation effect was not obvious. The concentrations of La(III) and Ce(III) in the organic phase after elution were lower than the concentrations of Pr(III) and Nd(III). The concentrations of La(III) and Ce(III) were 0.4% and 6.5% of the total rare earth concentration, respectively, while the concentrations of Pr(III) and Nd(III) accounted for 37.4% and 55.7% of the total rare earth ion concentration. However, the separation effect of La(III), Ce(III), Pr(III), and Nd(III) was obvious. The recovery rates of La(III), Ce(III), Pr(III), and Nd(III) under this cycle operation were 88.9%, 75.2%, 65.3%, and 98.0%, respectively. The recovery rates of Ce(III) and Pr(III) were only about 70%, indicating that the loss of rare earth was large during this step. The recovery rates of La(III) and Nd(III) were both about 90%, indicating that the loss was relatively small.

(7) The third small cycle of the organic phase after elution of the low-concentration rare earth mixed liquid system

**Figure 29:**
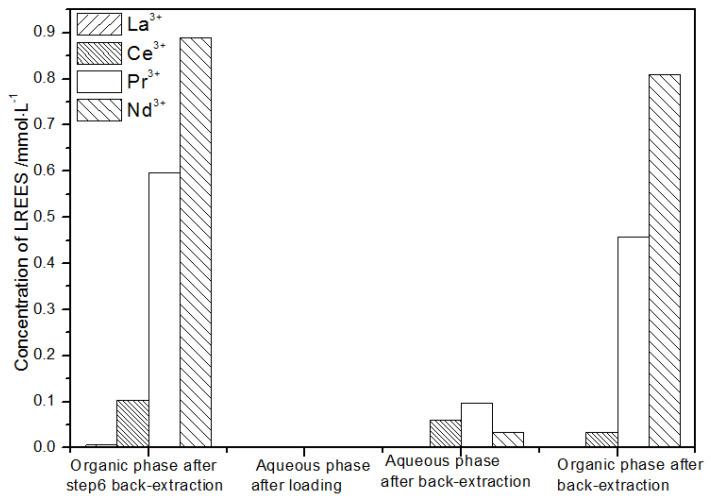
Concentration diagram of the third small cycle operation of the organic phase after elution of the low-concentration rare earth mixed liquid system.

After elution (see Figure 29), concentration, and loading, the eluted organic phase in step (6) was extremely low, indicating that almost all of the rare earth was contained in the TODGA organic phase. After back-extraction, the concentrations of Ce(III), Nd(III), Pr(III), and La(III) in the aqueous phase decreased in order. The concentration of La(III) was lower significantly, accounting for 1.3% of the total rare earth concentration. The concentrations of Ce(III), Pr(III), and Nd(III) accounted for 31.0%, 50.6%, and 17.2% of the total rare earth concentration, respectively, but the separation effect was not obvious. The concentrations of La(III) and Ce(III) in the organic phase after elution were lower than the concentrations of Pr(III) and Nd(III). The concentrations of La(III) and Ce(III) accounted for 0.2% and 2.6% of the total rare earth concentration, respectively, while the concentrations of Pr(III) and Nd(III) accounted for 35.1% and 62.0% of the total rare earth concentration, respectively. The separation effect of La(III) and Ce(III) with respect to Pr(III) and Nd(III) was obvious. The recovery rates of La(III), Ce(III), Pr(III), and Nd(III) were 91.7%, 90.6%, 93.1%, and 94.7%, respectively. Thus, the loss was relatively small.

When the low-concentration rare earth mixture was eluted by hydrochloric acid and nitric acid mixture, the back-extraction effect of rare earth separation and circulation operation in the aqueous phase was better than that in the organic phase. However, the rare earth recovery rate in the cyclic operation system of the rare earth separation step in the organic phase system was higher than that in the aqueous phase system.

## 4. Conclusions

In this study, *N*,*N*,*N*′,*N*′-tetraoctyl diglycolamide (TODGA) extractant was synthesized, and the optimal extraction conditions and the back-extraction characteristics of La(III), Ce(III), Pr(III), and Nd(III) by TODGA were studied. By controlling the number of back-extraction cycles and the concentrations of hydrochloric acid and nitric acid in the extraction system, the extraction and separation of mixed rare earth could be achieved.

Currently, the industrial extraction of REEs mainly involves 2-ethylhexyl phosphonic acid mono-2-ethylhexyl ester (P507) and di-2-ethylhexyl phosphate (P204), which exhibit effective separation and low cost; however, P507 and P204 can pollute the environment to some extent and, thus, are not ideal extractants [29,30]. The cost of TODGA is relatively low. Its degradation products are environmentally friendly and do not produce secondary pollution. It can be applied to the actual production, such that the current rare earth production process can become cleaner. It also has a wide range of application prospects.

## Data Availability

Not applicable.
